# The Evolution of Medicine*President’s address, Fifth District Medical Society.

**Published:** 1905-09

**Authors:** Malone Duggan

**Affiliations:** San Antonio, Texas


### TEXAS MEDICAL JOURNAL.

ESTABLISHED JULY, 1885.

PUBLISHED MONTHLY.—SUBSCRIPTION $1.00 A YEAR.

VOL. XXI. AUSTIN, SEPTEMBER, 1905. No. 3.

For Texas Medical Journal.

### The Evolution of Medicine.*

BY MALONE DUGGAN, M. D., SAN ANTONIO, TEXAS.

Whether or not the theory of evolution can be demonstrated as true, it serves as a necessary and acceptable hypothesis by which the origin and development of all life forms can be explained. Like the Atomic Theory in chemistry, it serves as a working basis for investigation and makes the explanation of natural growth intelligible. While limited in its sphere and failing to account for all that is of interest to man, it explains all that is knowable, and wisely omits from its operation that which is unknowable. But this omission is not a denial of the existence or operation of the knowable, which question, because of its very nature, is not susceptible of demonstration; but which operates independently of natural law, and is a necessary factor in the existence of man.

We will not review the first processes of this theory, when Matter and Force alone existed, and explain how these primary elements through countless ages evolved the terrestrial world; or how the outer crust of our own earth became modified and prepared for the habitation of life; or how, by natural selection, the various species and life forms were developed from one common protoplasmic cell. We will begin at a time when the species—man—became a conscious creature; when the moral element in him first began to assert itself. Then it was that natural selection ceased developing physical characters, and began the development of psychical characters; the moral and esthetic nature of man, which is the final process in the development of the species.

*President's address, Fifth District Medical Society.

And at this point, may the question be asked if it is not as rational to assume, that this new factor, introduced at this time, was not of Divine Nature, as it was to assume the existence of Matter and Force in the first instance? Neither assumption conflicts one with the other, but they both proceed in parallel lines to the accomplishment of one purpose. One is knowable and can be demonstrated; the other is unknowable and can not be demonstrated, but became necessary to the preservation of the species—Man. That must have been a purposive force that could have diverted the development of a species along physical lines into psychical channels; and that could have developed those mental qualities that so distinguish man from other life forms, more especially other species of the animal kingdom. Then back in the ages of the dim past man stood forth, equipped with hands and endowed with the faculty of judgment, entered the fight for existence; subject to the same unerring laws as was all other life forms. But thus armed he soon became master of his surroundings, and has since progressed, step by step, until his mighty intellect has all but solved the hidden secrets of nature.

As revealed in the record of fossils, the society of those days was primitive to the extreme; and interesting indeed is the history there recorded, showing how one generation after the other, moved from its own environment to a better one; and showing how energy expended, from the necessity of existence, produced a stimulus in the offspring, which resulted in a further endeavor and a movement into better environments. Thus it was that acquired characters became transmitted to succeeding generations; and in this manner developed society out of its crude state up to our modern civilization. But this advancement was not accomplished without hindrance or conflicting influences. Indeed, it was a heroic struggle, and long and fierce the conflict. Of necessity, medicine was the first practical custom to be developed out of this society. It would seem that some of the infirmities of man began early in the history of the race.

With these introductory remarks, it is our purpose to trace the progress of medical evolution from this beginning to the present time. So limited was the intellectual vision of these progenators of ours that very little progress was made for the first 5000 years. In fact, the first knowledge we have of the beginning of medicine was in Mythology. We are told that the goddess Isis, in the early days of Egypt, was the first practitioner of our art; and from these mythical legends we learn that all the nations of the earth, in this long period of time before the Christian era, contributed something to medical thought, especially Greece. It was a custom of the Babylonians to place their sick along the roadside, and to inquire of the passers by for their experience in similar conditions. Æsculapius, in the time of the Trogan wars, established what was called Æsculapicia, which continued through the many centuries, to serve both as hospital and temple, and were presided over by priests until the temples were closed by Constantine. It was believed that fire, air, water and earth constituted the primary elements of nature, and that the human body contained blood, phlegm, bile and black bile. When these constituents were disturbed or out of proportion, caused by some concoction or fermentation, disease was the result; and from the order and design observed about them, these ancients also concluded that the authorship of all things must have been some great spirit—Anima Mundi. From this understanding of the nature of things, it was a logical conclusion to attribute everything that concerned them to some god or gods; sickness, health, disaster, happiness, all were due to the wrath or pleasure of some offended or pleased god. To petition these deities for health and protection became a custom as the natural outcome of that belief; and from this practice there arose the necessity of an intermediator or priest, who, in time, regularly presided at the shrines of these deities, and in their priestly office they became physician as well. Thus in the beginning of our society, medicine and religion became inseparable, and continued so until within the last few centuries.

Hippocrates, contemporary with Pericles, Pythogoras, Socrates, Plato, Demosthenes and Thucydides, and influenced by their learning, became a great student; born in an Æscualpian temple, his vocation naturally turned to that of medicine. He soon developed an independence of thought, and began to search for natural causes in disease, and to apply deduction in his clinical observations. Thus he built up a system of medicine, and as he was the first to reduce his knowledge to writing, he has been rightly called the father of medicine. But, as he adhered to the same belief in the supernatural, and accepted the humeral doctrine of his predecessors, he made no real advancement in medical evolution.

Nor for the suceeding 500 years, until the time of Galen, was there any new principle added; although this was the period of Greece's greatest glory, the rise of the Alexanderian school and the triumphal ascendancy of Rome. During this period the sciences were fostered by Plato and Aristotle, and nourished by

Alexander the Great and the Ptolomies; and through the influence of Æsclepiades medicine science was established at Rome.

Galen came upon the scene at a time when the philosophy of Greece and Rome had done a great deal to elevate the intellectual faculty of man. He was learned in every department of knowledge of his time; and his criticisms of medical subjects were very thorough, and his deductions analytical. But without adding any new principle, and with the materials at his hands, he established a school of medicine, and so excellent was his work that his books remained as the accepted authority on medicine for 1000 years. And, on account of the permanent character his work gave to medicine, he must be regarded as a necessary link in its progress.

Following Galen's work there would have been a rapid and wonderful intellectual progress but for the influence of the church and the prieshood that became so powerful at that time. The teaching of Christ and His disciples was a new influence and inspiration; teaching, as it did, the love of man for man, it seized upon the life and thought of the people and soon became a mighty power. But, before a century had passed, ecclesiastical dogma had taken its place, and completely controlled the intellectual life. Without the knowledge of any natural laws, it was not unremarkable that all diseases should be ascribed to supernatural causes; and, accepting the doctrines of the church as of Divine Authority, no effort was made to discover any other causes. All diseases were attributed to the wrath of God, or to some evil spirit; and to be cured Divine Authority had to be invoked and suitable propitiation made for sins committed. And out of the miracles performed by Christ, for the cure of disease, there arose the belief in miracles, which became associated with some person or saint, from whom alone relief could be had. Any questioning of Divine authority was considered sacrilege, and as medicine used agencies independent of this authority, its practice became a reproach and was positively forbidden. Under this dominating influence further progress became effectually checked for fourteen centuries. However, the Jew, unprejudiced by any theological dogma, and the Arab, to whom no knowledge of it was known, the teachings of Hippocrates and Galen were kept alive through these dark ages, until medical schools were reintroduced into Europe by the followers of Mohammed. There were, also, in spite of ecclesiastical opposition, a few physicians who dared to practice their profession. In England, monastic schools were established in Oxford and London; and Charlemagne fostered learning in his empire. In the first few centuries Ætus of Constantinople, Alexander of Rome and Paulus of Ægus were very noted practitioners. But, when Justinia finally closed all medical schools in his empire, all physicians that did not embrace the Christian religion fled to Arabia, where all sciences were held in high esteem. There medicine was taught until the fourteenth century by Nestorius, Rhazes and Avicenna, who added much to practical medicine, and Geber, who developed, out of alchemy, some of the principles of modern chemistry.

Following the re-establishment of medical schools in Europe, notably in Spain and Italy, there began a revival of learning, resulting in the authority of the church being brought into question. The fourteenth, fifteenth and sixteenth centuries constituted a transitional period in which this independence of thought was developed. Anatomy was permitted again to be studied, and chemistry, for the first time, revealed some of nature's phenomena. The discoveries of Copernicus, Kepler and Galileo added much to strengthen this transition, and to turn the attention of investigators to the operation of natural laws. The world will never appreciate the cost of this struggle, or the character of the men who stood firm against the accepted dogma of that day. It was the making of a link in the chain of intellectual evolution, the importance is but now faintly appreciated. The men who thus bridged this long period of time, and who made our science possible, were most notably Mondino, Vesalius, Columbus, Eustachius, Fallopius, Ambrose Paré, Paracelus and Van Helmont.

The seed of independent thought and the spirit of investigation, planted by them, soon reaped the first harvest, early in the seventeenth century, when Harvey discovered the circulation of the blood. He demonstrated that blood, and not air, circulated through the veins, and thus overthrew the belief of over seventy centuries. This discovery can be truthfully considered the most important event in the evolution of the science. From this time on, medical investigations were conducted along new lines, and another great epoch was established in the chain of progress. So great was this influence that the spirit of investigation seized upon every branch of learning. Soon following Harvey's discovery was that of the lymphatic system, and the capillary circulation of the blood, made possible by the invention of the microscope. Later, Harvey also established his theory of generation. In chemistry, oxygen was discovered, followed soon by Dalton's atomic theory.

Investigation in physiology continued, and early in the eighteenth century Haller demonstrated his theory of muscular contractibility. He showed the contraction of muscular tissue to be independent of nerve, and distinct from sensibility. This theory was the final overthrow of the humeral doctrine of the ancients, and established in its stead the solodistic theory of Cullen and his colleagues. Cullen held that life was simply a state of excitation, and that when the stimuli was natural, health resulted, and when deficient in quality, or irritating, disease resulted. This theory served to explain the causation of disease for the following 100 years. In passing, it is but fair to mention that as a result of this progress in this period, electricity and galvanism were discovered, the obstetric forceps invented and that the itinerant physician ceased his wanderings, better medical schools were established, and the works of the Hunters, Desault, Bichart and others paved the way for the progress in anatomy and physiology that so rapidly followed. But the event of the greatest importance next to that of Harvey's discovery was that of the practical application of vaccination, in the prevention of smallpox by Jenner in the latter part of the eighteenth century, which was the key to the work of Pasteur, Koch and others, the result of which we all know so well.

Thus the dawn of the nineteenth century marked the beginning of a new epoch. The intellectual progress of the preceding few decades stimulated investigation, the ground was prepared to develop a harvest of new ideas and the opposition to scientific methods became less persistent. Early in the century the same methods of observation and investigation so effectually applied in developing physiology and anatomy were turned to the study of morbid conditions. Thus was developed pathology, which has aided so much in arriving at a knowledge of the true nature and cause of disease.

The invention of the stethoscope by Læennac added to the importance of diagnostic methods, and thus assisted materially in the continued evolution of medical thought.

Materia medica and therapeutics followed in this progressive temper, and early in the century the active principles of drugs were isolated and pharmacy assumed the importance it deserved.

Liebig, by his masterful researches in chemistry, and his demonstration of the fact that all organized bodies were composed of the primary elements of C. H. O. and N., or of the first three only, laid the foundation of organic chemistry, which, more than any other factor, has aided in forming our modern conceptions, and which is destined to accomplish still more in unraveling the mysteries of nature.

In nerve physiology, Bell, Hall and Miller demonstrated the motor and sensory columns of the spinal cord, and located the nerve centers for regulating the functions of the body in the medulla oblongata; also the motor areas in the brain were located. Thus many troublesome symptoms were explained and new light added to elucidate the nature of disease. And of equal importance was that of Andral's pathology of the blood. It was a demonstration of its physiological character, and introduced another new field for investigation. This new knowledge overthrew the solodistic theory of Cullen, which had stood for so long a time, and unlocked the gateway to the marvelous achievements in the line of physiologic chemistry, which is now considered with so much interest.

Along quite a different line of thought, but which has added the most to the achievement of medicine, and perhaps the most practical discovery the science will ever know, was that of anesthesia by Wells and Morton.

Soon after this great discovery Virchow demonstrated his cellular pathology, the modern basis of our science. With the improvement in the compound microscope, the gross anatomy was more minutely studied, with the resulting discovery that every structure in the body was formed by independent cells, which were composed of an organized substance, recognized as a physical basis of life, and having the characteristic property of multiplication and growth. From this knowledge of the human structures, which was found to apply equally to physiological and morbific conditions, we derive our present conception of disease.

But an understanding of the nature of disease did not explain its etiology. This was still a question of speculation; many theories were expounded, more or less igenious, but which failed to account for the true cause. But, with the idea that disease in some way was connected with miasma, and from an investigation of the soil and water, in the elucidation of this theory, bacteria were discovered. Then followed the work of Pasteur, Koch, Lister and others. Pasteur first demonstrated the relation of bacteria to fermentation, followed by Lister, who showed the connection of pathogenic bacteria to inflammation, which discovery alone made modern surgery possible. From these demonstrations it was but a step to ascribe bacteria as the cause of disease; and Koch soon discovered this fact in the case of tuberculosis, thus opening up that broad field which has placed the etiology of disease on a rational basis.

The knowledge of these facts has directed investigation along bio-chemical lines, with the result of producing specific sera and antitoxines, and their application in the treatment of disease. In this field we look forward to the next most important discoveries.

But there yet remains one important factor which is necessary to solve before etiology is thoroughly understood. For disease to result, there must be a specific cause and a predisposing influence. It is this condition of the system or immunity that now engages our most careful consideration. It is the final factor in the evolution of disease, and when fully comprehended will banish empiricism and establish itself on rational principles.

So rapid has been our progress along these lines, and so diffused has become learning in general, that it is necessary to specialize in every branch of medicine; and from now on we will look to the laboratory and the college specialist to lead us in new developments. To the general practitioners is left the important rôle of observing clinical conditions, and the application of laboratory methods to his practice. This is not the least important office, and, in proportion to the zeal and accuracy in which we practice our art, will be our measure of success. And this suggests another question which concerns us more directly than any other, and that is the correct interpretation of what is real progress and true science. As we have false philosophy, so we have pseudo-science; and the commercial element everywhere is trying to confuse the mind in this matter. It behooves us, therefore, to question well the authority of our medical literature, and to apply ourselves more assiduously to scientific study, to the end that we might become more discriminating.

In conclusion, I think it would be of interest to point out the fact that through all this long period of development, though paradoxical it may seem, it was the heretic that advanced our science—the brave men who dared to demonstrate their convictions in the face of the prevailing opinions of the times. In this process of evolution there was first an hypothesis assumed, then this was demonstrated, and, when finally accepted as a fact, society then advanced up to a higher plane of thought.

Again, as it was stated in the beginning of these remarks, science, particularly medicine, was inseparably associated with religion. It was seen from the beginning of society how man also believed in a spirit, and how this belief became responsible for the error in attributing disease to the wrath of an offended god.

Also how the teachings of Christ became misinterpreted by the church and checked learning for so many centuries. Now that the two have been completely divorced, one from the other, can we not state, in the spirit of fairness to truth, and for the good of the present, that while to science the credit is due for our present enlightenment, it, in return, owes a debt of gratitude to religion for the influence it has always exerted in the moral upbuilding of man, thereby preparing the way for the acceptance and growth of scientific truth. Now and in the future both factors will be explained, one as interpreting natural laws and the other spiritual laws, both operating to the same purpose—the welfare of mankind.

